# White Matter Changes in Patients with Amnestic Mild Cognitive Impairment Detected by Diffusion Tensor Imaging

**DOI:** 10.1371/journal.pone.0059440

**Published:** 2013-03-21

**Authors:** Jianghong Liu, Changhao Yin, Shugao Xia, Longfei Jia, Yanqin Guo, Zhilian Zhao, Xiaobo Li, Ying Han, Jianping Jia

**Affiliations:** 1 Department of Neurology, Xuanwu Hospital, Capital Medical University, Beijing, PR China; 2 Key Neurodegenerative Laboratory of Ministry of Education of the People’s Republic of China, Beijing, PR China; 3 Department of Neurology, Hongqi Hospital of Mudanjiang Medical College, Mudangjiang, China; 4 Gruss Magnetic Resonance Research Center, Departments of Radiology, Neuroscience, Psychiatry and Behavioral Sciences, Albert Einstein College of Medicine, Yeshiva University, Bronx, New York, United States of America; 5 Department of Neurology, Tongren Hospital, Capital Medical University, Beijing, China; 6 Department of Radiology, Xuanwu Hospital, Capital Medical University, Beijing, China; University of Manchester, United Kingdom

## Abstract

Compared to normal aging adults, individuals with amnestic mild cognitive impairment (aMCI) have significantly increased risk for progressing into Alzheimer’s disease (AD). Autopsy studies found that most of the brains of aMCI cases showed anatomical features associated with AD pathology. The recent development of non-invasive neuroimaging technique, such as diffusion tensor imaging (DTI), makes it possible to investigate the microstructures of the cerebral white matter *in vivo*. We hypothesized that disrupted white matter (WM) integrity existed in aMCI. So we used DTI technique, by measuring fractional anisotropy (FA) and mean diffusivity (MD), to test the brain structures involved in patients with aMCI. DTI scans were collected from 40 patients with aMCI, and 28 normal controls (NC). Tract-based spatial statistics (TBSS) analyses of whole-brain FA and MD images in each individual and group comparisons were carried out. Compared to NC, aMCI patients showed significant FA reduction bilaterally, in the association and projection fibers of frontal, parietal, and temporal lobes, corpus callosum, bilateral corona radiation, right posterior thalamic radiation and right sagittal stratum. aMCI patients also showed significantly increased MD widespreadly in the association and projection fibers of frontal, parietal and temporal lobes, and corpus callosum. Assessment of the WM integrity of the frontal, parietal, temporal lobes, and corpus callosum by using DTI measures may aid early diagnosis of aMCI.

## Introduction

Mild cognitive impairment (MCI) is a descriptive category that identifies patients with memory impairment beyond that expected for age and education, who do not qualify for a diagnosis of dementia [1]. The term MCI has become widely used, with the suggestion that it is a prodromal state of Alzheimer’s disease (AD) and possibly other dementias [2], [3].

MCI has been classified broadly into amnestic and nonamnestic subtypes. Amnestic MCI (aMCI) presents clinically with memory disturbance as the main feature, although other cognitive domains may be affected as well. aMCI is thought to progress into AD, whereas nonamnestic MCI may progress to the other types of dementia [4]. Individuals with aMCI have heightened risk of progressing to AD [5]. Recent autopsy studies indicate that AD pathology associated anatomical features were found in most of the brains from aMCI patients [6], [7]. A meta-analysis reported the annual conversion rate from aMCI to AD at 5%–10% [8].

Since individuals with aMCI have increased risk of progressing to AD, it is important to appropriately diagnose aMCI and identify features that would predict an early progression to AD. Biomarkers reliably identifying those aMCI patients who are most at risk of progressing to AD would therefore be useful, especially as preventive interventions become available. Diffusion tensor imaging (DTI) may be one such technique. DTI is a quantitative MRI technique that measures the movement of water within the tissue microstructure [9]. Loss of anisotropic diffusion could be related to abnormalities within the cellular microstructure, which provide information about disrupted structural integrity in that area. The integrity of white matter (WM) fiber bundles in the nervous system can be evaluated by fractional anisotrophy (FA) and mean diffusivity (MD) measures [10], [11], [12].

Reduced FA values were reported in WM structures from the temporal and parietal lobes and posterior cingulum bundle in patients with aMCI, compared to controls [13], [14], [15], [16], [17]. In addition, compared to normal aging adults, widespread WM structural abnormalities have been found in patients with AD, including reduced FA and increased MD within the splenium of the corpus callosum [18], [19], [20], [21], [22], the cingulum bundle [14], [17], [23], [24], [25], [26], and cortical regions [13], [15], [18], [20], [26], [27].

Despite of the advances, a discrepancy exists among the findings of the previous DTI studies of AD and aMCI. To give a comprehensive view of the degeneration patterns of the WM in aMCI patients, we used the newly developed tract-based spatial statistics (TBSS) method and multiple diffusion measures (FA and MD) to systematically study aMCI-associated changes in WM tracts across the whole brain. The present study included 40 patients with aMCI and 28 demographically-matched normal control (NC) participants. We hypothesized that WM anomalies in corpus callosum and regions connecting the posterior cingulum and limbic system may be the neurobiological marker of aMCI.

## Subjects and Methods

### Subjects

Patients with aMCI were recruited from Beijing Xuanwu Hospital of Capital Medical University in China, from January 2009 to December 2011. Normal controls were recruited from local residents of Beijing Xuanwu District. The study was approved by the institutional review board of Beijing Xuanwu Hospital. Written informed consents were obtained from all participants. The patient group included 40 subjects diagnosed with aMCI (age 65.7±7.2 years; 17 males and 23 females; mean education duration 10.3±4.3 years) according to Petersen’s diagnostic criteria [1]. The aMCI patients had the ability to understand and write the informed consents. The NC group included 28 subjects who were cognitively normal and had a Clinical Dementia Rating (CDR) of 0 [28], [29] (63.5±7.2 years; 11 males and 17 females; mean education duration10.5±4.1 years). All participants underwent a series of neurological tests and a battery of neuropsychological assessments, which included the Montreal cognitive assessment (MoCA) [30] and the Clinical Dementia Rating Scale (CDR) [29].

There were no demographic differences between the groups with regards to age, sex and education. In addition, the prevalence of vascular factors such as hypertension, hypercholesterolemia, and heart attack, did not differ between the groups. The subjects had no history of a psychiatric or neuropsychological disease.

### DTI Acquisition Protocol

DTI data was acquired from each subject on a Siemens 3.0 Trio Tim MRI system with cranial 12-channel phased array surface coil using an echo planar imaging (EPI) sequence in 32 independent, non-collinear directions of a b-value = 1000 s/mm^2^, and one additional image with no diffusion weighting (b = 0). TR = 11000 ms, TE = 98 ms, flip angle = 90°, field of view (FOV) = 256 mm×256 mm, imaging matrix = 128×128, number of slices = 60, and slice thickness = 2 mm. Three acquisitions were averaged to increase the signal-to-noise ratio.

### Data Processing

DTI data processing was carried out using FSL software (FMRIB Software Library, http: //www.fmrib.ox.ac.uk/fsl) [31]. Initially, eddy current correction was run to correct gradient-coil distortions and small-head motions using affine registration to a reference image (b0 volume) [32]. The brain voxels of DTI data were extracted using the Brain Extraction Tool (BET) [33]. The maps of diffusion tensor parameters including FA and MD were calculated using DTI-FIT tool, which fits a diffusion tensor model to diffusion-weighted images for each voxel.

Voxel-wise statistical analysis of the FA and MD data was performed for regional differences using TBSS [34], which has been reported to be more precise than conventional VBM style analysis. Firstly, images from all individuals were aligned into a 1 × 1 × 1 mm^3^ Montreal Neurological Institute (MNI) 152 space using the nonlinear registration (b-spline representation of the registration warp field). Next, a mean FA image of all aligned FA images was calculated and thinned to create the mean FA skeleton, which represents the centers of all tracts common for the group. The skeleton's threshold was FA ≥0.20 to ensure that areas of low FA and/or high inter-subject variability were excluded from the analyses. Finally, each subject's aligned FA data was projected onto the skeleton by searching perpendicular to the local skeleton structure for the maximum value in the subject’s skeletonized FA image, and the resulting data was fed into voxel-wise cross-subject statistics. The John Hopkinson University (JHU) white-matter atlas was applied to identify the names of WM bundles that contained the clusters of significant between-group FA differences.

### Statistical Analysis

Group statistical analysis was then conducted only on voxels within the white-matter skeleton mask. Differences in FA and MD between aMCI group and normal control were assessed using voxel-wise two-sample *t* -tests. Nonparametric permutation tests and Threshold-Free Cluster Enhancement (TFCE) option were conducted based on 5,000 random permutations. The clusters with a TFCE-corrected P-value of less than 0.05 or 0.0001 were reported. The fiber tracts and their corresponding clusters were identified using the Johns Hopkins University DTI-based White Matter Atlas [35].

Demographic data was analyzed by using two-sample *t*-test.

## Results

### Demographic Information

As shown in **Table 1**, there were no significant demographic differences between the two groups.

**Table 1:** Demographic Characteristics of both groups.

251660288	aMCI (n = 40) (mean ± SD)	NC (n = 28) (mean ± SD)	Test statistic	P
Age	65.7±7.2	63.5±7.2	t = 1.21	0.23
Male/Female	17/23	11/17	*χ^2^* = 3.60	0.06
Education	10.3±4.3	10.5±4.1	t = 0.25	0.80
CDR	0.5	0	t = 12.6	<0.00001
MoCA [67]	20.8±3.95	26.3±2.77	t = −6.33	<0.00001

### Reduction of FA in aMCI Patients

**Figure 1:**
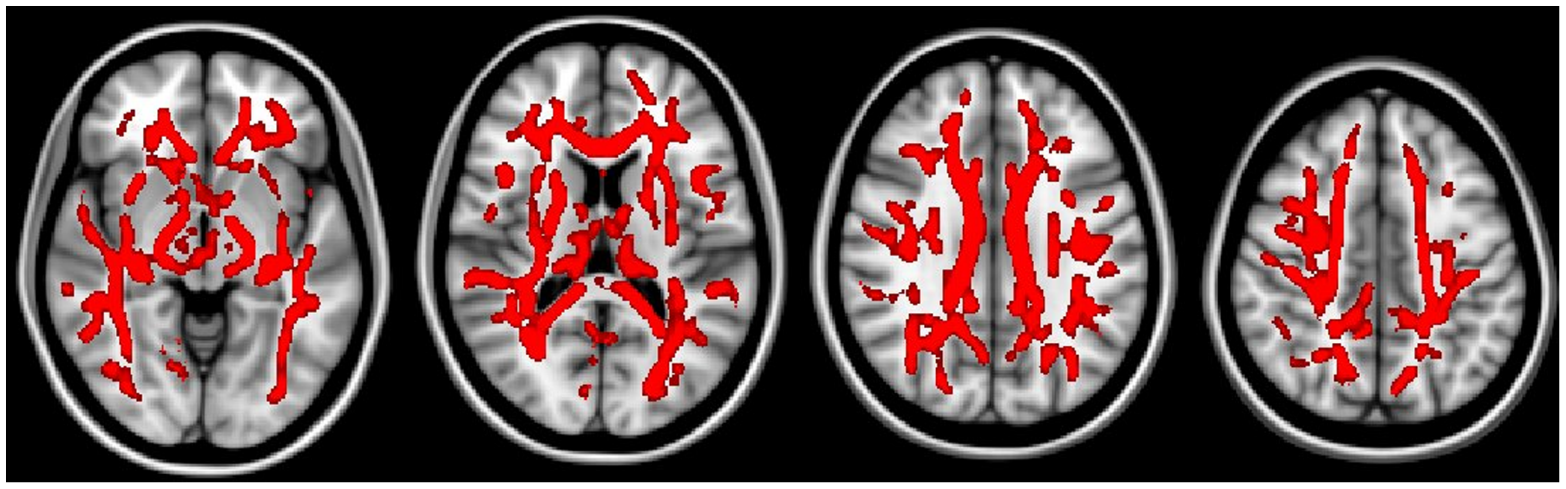
The regions having significantly reduced FA values in the aMCI group compared to the control group (TFCE corrected, p<0.05).

**Figure 2:**
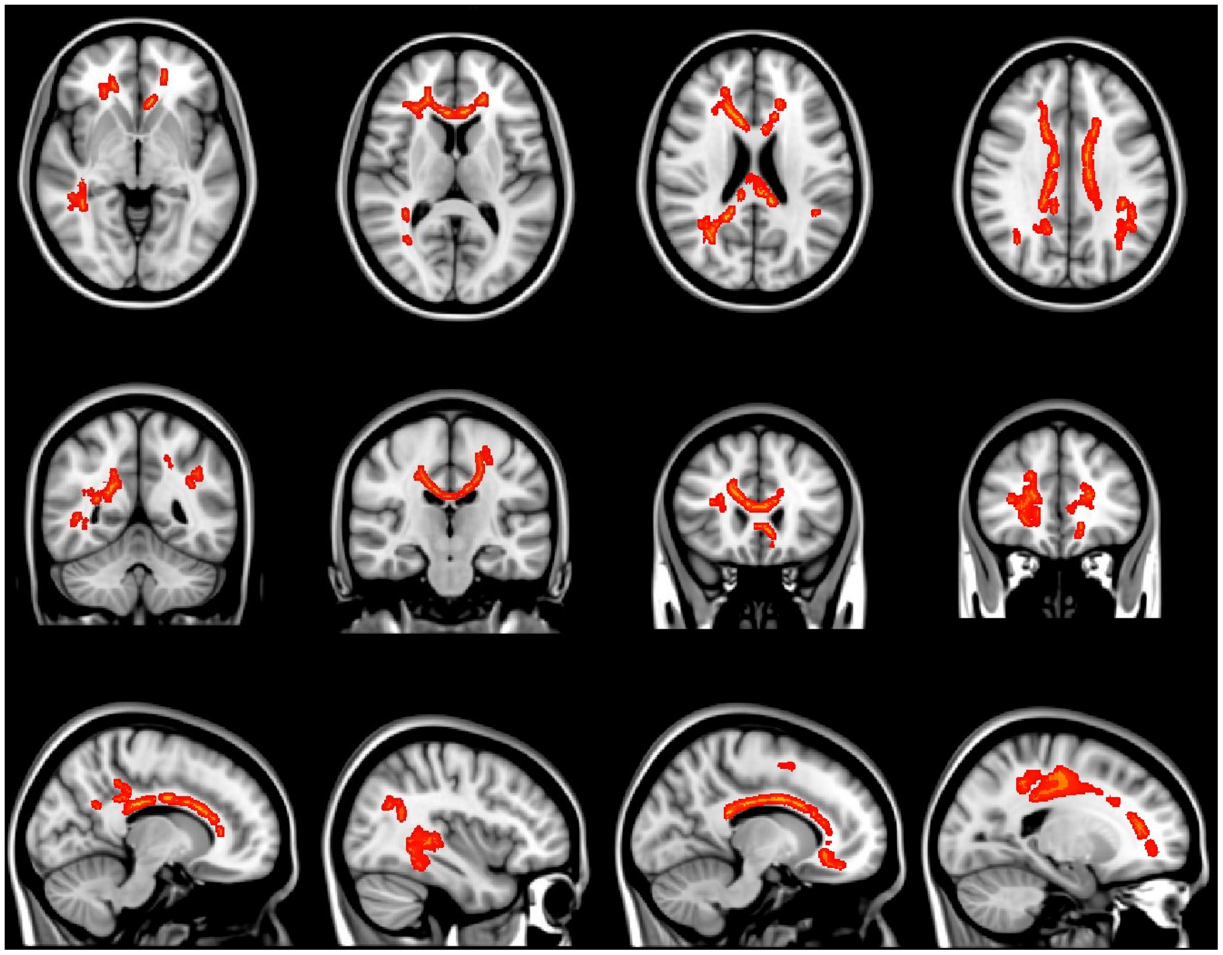
The regions having significantly reduced FA values in the aMCI group compared to the control group (TFCE corrected, p<0.0001).

Between-group comparisons of the voxel-wise FA values showed that patients with aMCI exhibited significantly reduced FA values (TFCE-corrected *p*<0.05) in WM clusters over large areas bilaterally, mainly in the frontal, parietal, and temporal lobes, the whole corpus callosum, and its association fibers including the superior longitudinal fasciculus, external capsule, cingulum bundle, sagittal stratum and fornix, projection fibers including internal capsule, corona radiation, and thalamic radiation (**Figure 1**). The clusters with even higher between-group differences (with a TFCE–corrected significance of *p*<0.0001) were observed in regions of corpus callosum, bilateral corona radiation, right posterior thalamic radiation and right sagittal stratum (**Figure 2** and **Table 2**). We did not find brain clusters with significantly increased FA values in the aMCI group, compared to the normal controls.

**Table 2:** The anatomical areas that showed significantly reduced FAvalues in aMCI compared to normal controls (TFCE- corrected, p<0.0001).

Anatomical region	MNI coordinates (mm)			Cluster size
	x	y	z	
Corpus callosum	10	18	21	2292
Corona radiation R	18	15	30	1131
Corona radiation L	−18	−25	35	694
Posterior thalamic radiation R	37	−42	4	220
Sagittal stratum R	40	−38	−11	118

### Increase of MD in aMCI Patients

**Figure 3:**
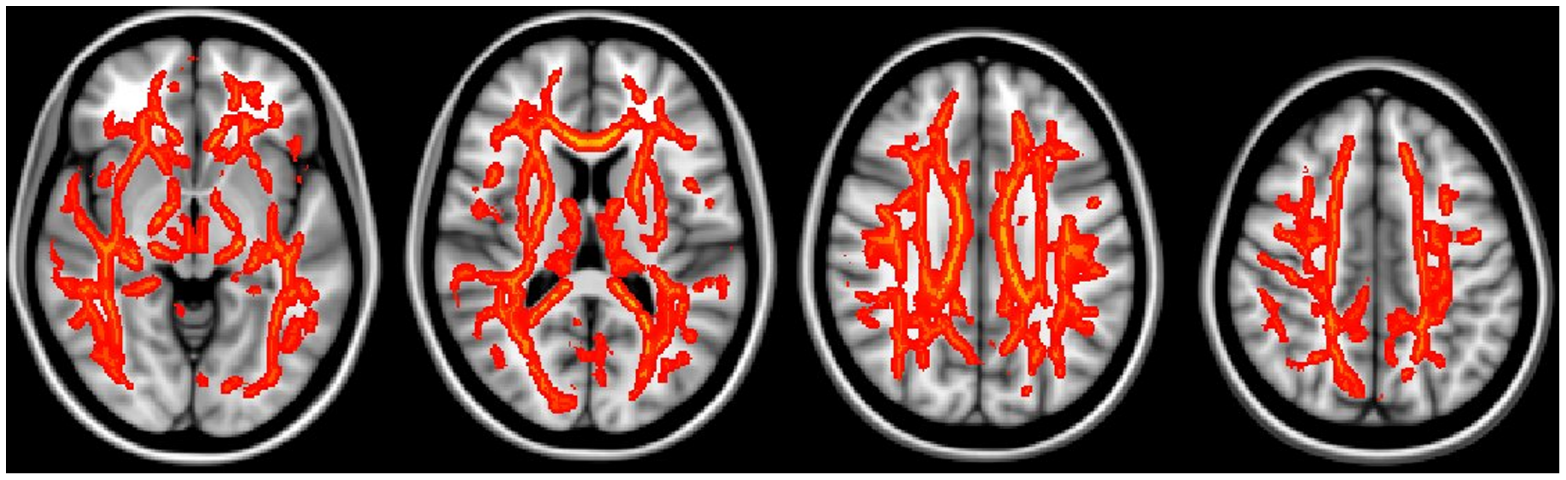
The regions having significantly increased MD values in the aMCI group compared to the control group (TFCE corrected, p<0.05).

**Figure 4:**
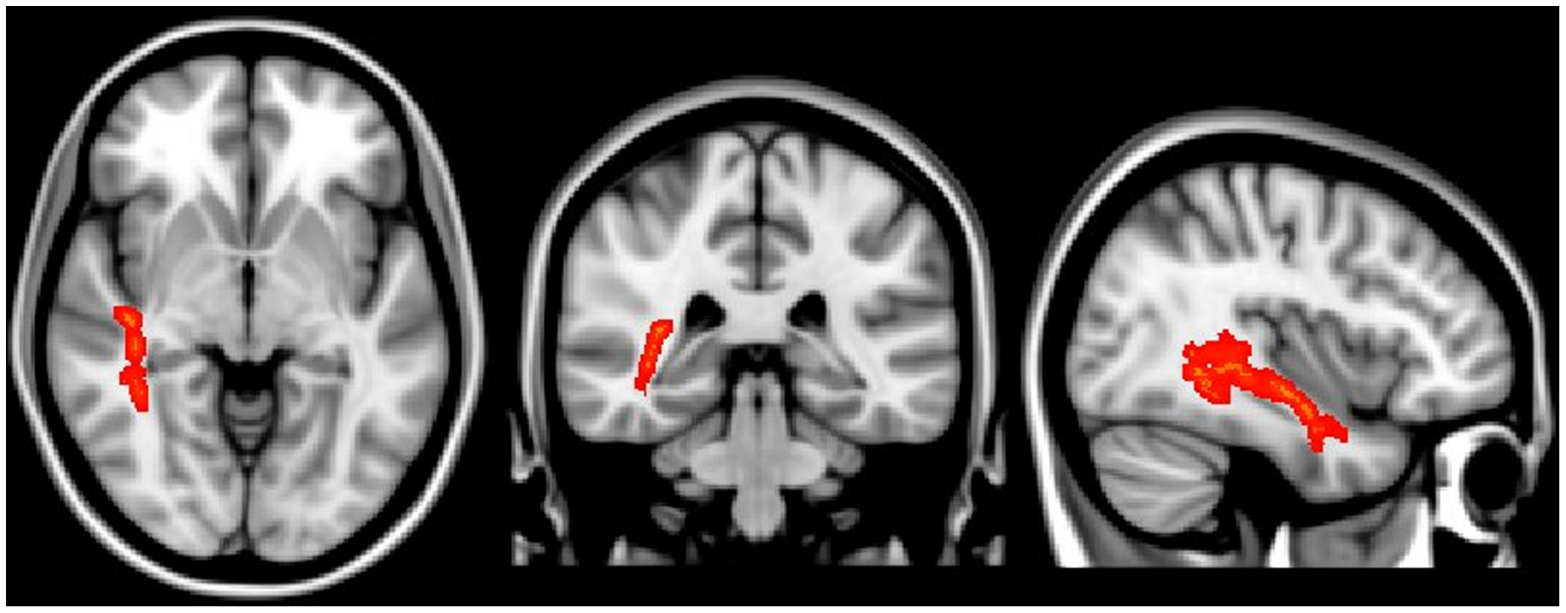
The regions having significantly increased MD values in the aMCI group compared to the control group (TFCE corrected, p<0.0001).

Between-group comparisons of the voxel-wise MD values showed that patients with aMCI exhibited significantly increased MD values (TFCE-corrected *p*<0.05) in WM clusters bilaterally, especially in the frontal, parietal, and temporal lobes, corpus callosum, association fibers (including the superior longitudinal fasciculus, external capsule, cingulum bundle, sagittal stratum), projection fibers (including internal capsule, corona radiation, thalamic radiation) (see details from **Figure 3**). The clusters with even higher between-group differences (with a TFCE–corrected significance of *p*<0.0001) were observed in the right Retrolenticular part of internal capsule, right posterior thalamic radiation and right sagittal stratum (**Figure 4** and **Table 3**). We did not find brain clusters with significantly decreased MD values in the aMCI group, compared to the normal controls.

**Table 3:** The anatomical regions that showed significantly increased MD values in aMCI compared to normal controls (TCFE-corrected, p<0.0001).

Anatomical region	MNI coordinates (mm)			Cluster size
	x	y	z	
Retrolenticular part of internal capsule R	39	−33	−1	66
Posterior thalamic radiation R	38	−50	3	65
Sagittal stratum R	39	−15	−10	154

## Discussion

Since aMCI is a high-risk status for developing AD, it is important to appropriately diagnose aMCI and identify features that would predict an early progression to AD. By comparing the FA and MD from DTI data, the present study indicated that, in aMCI patients, the FA and MD were significantly changed over large brain areas. These results suggest that FA and MD can reflect the pathological features of aMCI patients and may be considered as a tool for the early prediction of aMCI.

A recent study found that FA in the left frontal superior gyrus and MD in the genu of the corpus callosum, left anterior cingulated gyrus, and right corona radiata emerged as the best combination for predicting aMCI [36]. Our results are consistent with previous studies which have reported changes in anterior and posterior cingulate, corpus callosum, and frontal lobe [13], [14], [17], [37]. Besides these, we observed increased MD in subcortical regions such as the right posterior thalamic radiation and internal capsule. The internal capsule and corona radiata contain projection fibers traveling from the cortices to the brainstem and from the thalamus to the cortices. These regions are supplied by penetrating arteries and thus are susceptible to ischemic damage. Further studies are needed to determine the contribution of vascular disease and risk factors to changes in the basal ganglia and corona radiata.

Decreased FA in the corpus callosum has been seen in AD [38]. Gray matter (GM) atrophy has been observed in parietal and temporal lobes, posterior cingulate, and hippocampus in aMCI [39], [40], [41]. Other studies also indicated GM atrophic change in the hippocampus of aMCI brain [42], [43]. Therefore, hippocampal atrophy and disruption of hippocampo-parieto-frontal WM network are thought as the key pathogenesis of AD and aMCI [44]. Both AD and aMCI patients suffer gradually impaired memory [45], which is highly associated with the disturbance of hippocampal synaptic plasticity that is modulated by multiple molecules and mechanisms [46], [47]. Although the present study did not observe significant change of FA and MD around hippocampus, we did find change of the frontal and parietal WM as well as frontal WM and tracts connecting with limbic system, suggesting that WM disturbance may be independent of GM changes in aMCI patients [48].

Teipel et al. [49] showed decreased functional connectivity in posterior regions in aMCI using combined EEG and DTI. Some studies have found especially large reductions in FA in parahippocampal regions [17], [26], [50]. Furthermore, another study proved the association between parahippocampal WM change and declarative memory performance in aMCI patients [51], while the present study did not detect change in parahippocampal WM but did detect changes in many associate fibers and projecting fibers. These inconsistence may be resulted from different methods and standardization. Ward et al [52] suggested standardized definition and better techniques for aMCI and AD was needed.

Previous studies have found changes in the anterior cingulate in aMCI using DTI [36]. A more recent study found that aMCI participants showed lower posterior cingulated (PC) WM integrity relative to those with non-aMCI. Findings implicate involvement of posterior microstructural WM degeneration in the development of MCI-related cognitive changes and suggest that reduced FA of the PC may be a candidate neuroimaging marker of AD risk [53]. In Delano-Wood L’s study, both non-aMCI and aMCI subgroups showed degradation of the PC, although those with aMCI showed more profound changes in this region coupled with additional changes to the splenium and parahippocampal white matter. In our study, we did not find difference of PC between aMCI and NC. The variation may be due to differences in the study population and method of DTI analysis. Future tractography studies may be able to determine the extent of changes more reliably in both the anterior and posterior cingulate.

We observed changes in almost the whole corpus callosum. But the mechanisms of the changes are not the same. The changes in the splenium of the corpus callosum might because of Wallerian degeneration. On the other hand, the genu of the corpus callosum is known to myelinate much later in development when compared to other WM regions, and the changes in the genu of the corpus callosum in aMCI patientsof our results might support the retrogenesis hypothesis [54] which posits that later-myelinating fibers (e.g., limbic pathways such as the cingulum, see from [55], are more susceptible to myelin breakdown than earlier myelinating fibers (e.g., posterior limb of the internal capsule). So it seems plausible to affirm that both Wallerian degeneration and myelin breakdown mechanisms are responsible for the region-specific illness effects. In this view, Wallerian degeneration affects the posterior corpus callosum subregion that receives axons directly from those brain areas (temporo-parietal lobe regions) that are primarily affected by AD pathology. Differently, the myelin breakdown process might affect the later myelinating corpus callosum subregion, causing changes in the genu of the corpus callosum [56]. In addition, Agosta and collages found aMCI patients only showed increased axial diffusivity in tracts projecting to the frontal cortex and splenium of the corpus callosum [57], which is consistent with present study and previous study [58] showing significant change of corpus callosum, and suggests the importance of corpus callosum in aMCI patients. By the approach of TBSS, Preti et al detected voxels with a statistically significant FA reduction in every corpus callosum portion of aMCI patients compared to health control [59]. We tested the hypothesis that both Wallerian degeneration and myelin breakdown might be responsible for the region-specific callosal change in aMCI patients and the results were coincidence with those of the mild AD patients in a VBM and DTI study [60] which suggest that both mechanisms affecting the callosal WM are present. Overall, all of these findings suggest that damage of corpus callosum may be a significant feature of aMCI.

Sperling et al. [61] suggested that, WM changes may be initiated through the accumulation of amyloid-β which may subsequently trigger the release and activation of various pro-inflammatory neurotoxic substances and microglia [62], [63]. Although amyloid plaques and neurofibrillary tangle formation have been considered as two hallmarks of AD [45], they are not so easy to be detected by non-invasive methods, particular in the early aMCI stage. Recently, DTI-based FA and MD measures are considered useful for predicting disease progression. It was reported that FA, MD and hippocampal volumes were good predictors of progression of AD, with likelihood ratios >83, and more than 90% accuracy [64]. In another study, MD was found to be correlated positively with the atrophy level in aMCI patients in the right temporal portion of the uncinate, middle longitudinal and inferior longitudinal fasciculi and in the parathalamic WM, the fornix and the posterior cingulum of the right hemisphere; whilst, FA was associated with atrophy in all WM areas except the middle longitudinal fasciculus [65]. Furthermore, it was reported that DTI had a sensitivity of 80% and specificity of 60.3% in distinguishing aMCI from non-aMCI and the normal group [66]. These studies indicated that FA and MD from DTI data are two indices with significant values in early diagnosis of aMCI. Of course, there were some inconsistentce about it. For example, it was found that although there was a significant increase of MD in parahippocampal WM but no difference of FA [51]. And there were also inconsistency about the changes of FA and MD in different brain area [52]. These inconsistence remains to be elucidated by further study using more sophisticated techniques.

The findings of the present study suggest that FA reduction and/or MD increase in the cingulum bundle may be a reliable marker of the onset of aMCI, and the progressing of the disrupted cingulum bundle WM integrity could be useful for assessing the progressing of the disease from aMCI into AD. To validate these hypotheses, the results of this study need to be replicated in a larger sample. Longitudinal studies are needed to follow-up the clinical status of the participants involved in this study, and to examine whether the participants, who developed into AD from aMCI, would have significantly severe and faster cingulum bundle WM anomalies, compared to the WM status in other brain regions in the same subjects with AD and the whole brain WM status in those who did not develop into AD.
